# SARS-CoV-2 Impairs Osteoblast Differentiation Through Spike Glycoprotein and Cytokine Dysregulation

**DOI:** 10.3390/v17020143

**Published:** 2025-01-22

**Authors:** Rosa Nicole Freiberger, Cynthia Alicia Marcela López, Patricio Jarmoluk, María Belén Palma, Cintia Cevallos, Franco Agustin Sviercz, Tomás Martín Grosso, Marcela Nilda García, Jorge Quarleri, M. Victoria Delpino

**Affiliations:** 1 Consejo Nacional de Investigaciones Científicas y Tecnológicas (CONICET), Laboratorio de Inmunopatología Viral, Instituto de Investigaciones Biomédicas en Retrovirus y Sida (INBIRS), Universidad de Buenos Aires (UBA), Buenos Aires 1121, Argentina; freibergernicole@gmail.com (R.N.F.); alilopez1996@gmail.com (C.A.M.L.); patriciojarmoluk@gmail.com (P.J.); cevalloscintia@gmail.com (C.C.); francosviercz@gmail.com (F.A.S.); grossotomas@gmail.com (T.M.G.); quarleri@fmed.uba.ar (J.Q.); 2 Cátedra de Citología, Histología y Embriología, Facultad de Ciencias Médicas, Universidad Nacional de La Plata, La Plata 1900, Argentina; mbpalma@med.unlp.edu.ar (M.B.P.); mngarcia@med.unlp.edu.ar (M.N.G.); 3 Laboratorio de Investigación Aplicada a Neurociencias (LIAN), Fleni, Consejo de Investigaciones Científicas y Técnicas (CONICET), Escobar 1625, Argentina

**Keywords:** COVID-19, SARS-CoV-2, bone, osteoblasts

## Abstract

Pulmonary and extrapulmonary manifestations have been reported following infection with SARS-CoV-2, the causative agent of COVID-19. The virus persists in multiple organs due to its tropism for various tissues, including the skeletal system. This study investigates the effects of SARS-CoV-2 infection, including both ancestral and Omicron viral strains, on differentiating mesenchymal stem cells (MSCs), the precursor cells, into osteoblasts. Although both viral strains can productively infect osteoblasts, precursor cell infection remained abortive. Viral exposure during osteoblast differentiation demonstrates that both variants inhibit mineral and organic matrix deposition. This is accompanied by reduced expression of runt-related transcription factor 2 (RUNX2) and increased levels of interleukin-6 (IL-6), a cytokine that negatively regulates osteoblast differentiation. Furthermore, the upregulation of receptor activator of nuclear factor kappa B ligand (RANKL) strongly suggests that the ancestral and Omicron variants may disrupt bone homeostasis by promoting osteoclast differentiation, ultimately leading to the formation of bone-resorbing cells. This process is dependent of spike glycoprotein since its neutralization significantly reduced the effect of infective SARS-CoV-2 and UV-C inactivated virus. This study underscores the capacity of ancestral and Omicron SARS-CoV-2 variants to disrupt osteoblast differentiation, a process essential for preserving the homeostasis and functionality of bone tissue.

## 1. Introduction

The emergence of Coronavirus Disease 2019 (COVID-19), caused by the severe acute respiratory syndrome coronavirus 2 (SARS-CoV-2), has reshaped global health priorities, often progressing to severe pneumonia in many patients [1]. In regions with limited healthcare resources and a high prevalence of chronic illnesses, the rising case numbers remain a pressing challenge. While many countries have transitioned into the post-epidemic phase, the need for close clinical monitoring of recovering patients has never been more critical.

The skeleton is a dynamic organ system that undergoes continuous remodeling through the coordinated actions of osteoblasts and osteoclasts. Osteoclasts facilitate bone resorption through acidification and the secretion of lysosomal enzymes. In contrast, osteoblasts are responsible for the deposition of bone matrix and are thought to facilitate the calcification and mineralization of the bone matrix [2]. This intricate process involves the deposition and mineralization of the bone matrix, both of which are essential for strengthening and maintaining the skeleton. A key player in facilitating mineralization is alkaline phosphatase (ALP) [3,4]. Type I collagen, the primary organic component of bone, constitutes 90% of the organic matrix [5]. The proper deposition of the organic matrix is crucial for aligning the mineral matrix precisely, thereby ensuring the structural integrity and functionality of bone [6]. Furthermore, evidence suggests that both the disease and its treatments exert lasting negative effects on the bone health of survivors, particularly among the elderly and frail. This raises concerns about heightened risks of bone loss in these vulnerable populations [7]. Intriguingly, research shows that the extent of bone loss—whether local or systemic—aligns closely with the severity of the inflammatory response. Alarmingly, this inflammation-induced bone loss can persist even after successful treatment and resolution of the primary infection [8]. Recent findings support this connection, showing that reduced vertebral bone mineral density (BMD) is associated with worse outcomes in COVID-19 patients, underscoring its potential as a key marker of disease severity [9]. Furthermore, preclinical studies have shed additional light on the harmful impact of SARS-CoV-2 on bone and muscle health [10].

We have recently started to identify how SARS-CoV-2 infection causes bone damage. Our findings demonstrated that ancestral and Omicron variants of SARS-CoV-2 induce an increase in osteoclast numbers, leading to excessive bone resorption [11]. Currently, it remains unexplored whether SARS-CoV-2 infection affects osteoblast differentiation and function. This study explores the direct effects of SARS-CoV-2 on osteoblast differentiation and function, using mesenchymal stem cells (MSCs) derived from human umbilical cords as precursor cells. The findings demonstrate that SARS-CoV-2 exposure inhibits osteoblast differentiation. These results reveal a novel mechanism through which SARS-CoV-2 may directly impair bone health, potentially contributing to long-term skeletal consequences in COVID-19 survivors.

## 2. Materials and Methods

### 2.1. Isolation and Expansion MSCs

Umbilical cords were preserved in α-MEM and processed according to previously described methods [12]. Each umbilical cord was cut into 5 mm fragments, and a sagittal incision exposed Wharton’s jelly. Umbilical blood vessels were carefully removed with clamps. The fragments were washed 2–3 times with Dulbecco’s phosphate-buffered saline (Sigma-Aldrich, St. Louis, MI, USA) to remove residual blood. The exposed jelly section was placed face down in a culture plate with α-MEM and 10% platelet lysate. Plates were incubated at 37 °C in a humid, 5% CO_2_ atmosphere, with medium changes every 2–3 days. Umbilical cord-derived MSCs expansion was observed within 10–14 days and continued until passage 2/3. MSCs were characterized by the expression of CD105, CD73, and CD90, and the absence of CD45, CD34, CD14, CD19, and HLA-DR [13]. For experiments, MSCs were cultured in α-MEM supplemented with 10% heat-inactivated fetal bovine serum (Gibco-BRL, Life Technologies, Grand Island, NY, USA), 100 U/mL penicillin, and 100 mg/mL streptomycin (complete medium) and used up to passage 5. This study was approved by the Comité de Bioética y Ética de la Facultad de Ciencias Médicas de la Universidad de Buenos Aires, Argentina (RESCD-2024-429E). Written informed consent was obtained from each mother before cesarean birth, and human umbilical cords were obtained from discarded placentas.

### 2.2. Osteoblast and Adipocyte Differentiation from MSCs

Mesenchymal stem cells (MSCs), the precursor cells for both osteoblasts and adipocytes, were initially seeded at a density of 5 × 10^4^ cells per well in 24-well plates in α-Minimal Essential Medium (α-MEM, Gibco, Waltham, MA, USA) containing 2 mM L-glutamine, 10% fetal bovine serum (FBS) (Gibco), 100 U/mL penicillin, and 100 μg/mL streptomycin (complete medium). Osteoblast differentiation medium (complete medium plus 10 mM β-glycerophosphate, 0.1 µM dexamethasone, and 50 µM ascorbic acid, all from Sigma), achieving complete differentiation by days 14–21. Alternatively, adipocyte differentiation medium (α-MEM containing 2 mM L-glutamine, 10% FBS (Gibco), 100 U/mL penicillin, and 100 μg/mL streptomycin, and supplemented with 0.5 mM 3-isobutyl-1-methylxanthine [IBMX], 0.01 µM dexamethasone, and 10 µg/mL human insulin, all from Sigma Aldrich) was used, achieving complete differentiation within 7–10 days.

### 2.3. Viral Infection of Osteoblasts, Adipocytes, and MSCs

Dr. Sandra Gallego kindly provided the ancestral SARS-CoV-2 strain (Wh) from the Universidad Nacional de Córdoba, Argentina. The Omicron (BA.5) strain was isolated from a nasopharyngeal swab, characterized, and propagated in Vero E6 cells. Both strains were subsequently titrated, achieving a titer of 2.85 × 10⁶ TCID₅₀/mL.

Vero E6 cells from American Type Culture Collection (ATCC) were cultured in Dulbecco’s Modified Eagle Medium (DMEM, Sigma-Aldrich) supplemented with 2 mM L-glutamine, 10% FBS, 100 U/mL penicillin, and 100 μg/mL streptomycin, and maintained at 37 °C in a 5% CO₂ atmosphere.

For infections, osteoblasts and MSCs were exposed to the virus at multiplicities of infection (MOI) of 0.1 and 1.0. The infection process involved a 4 h incubation in α-MEM without FBS, followed by 4–5 washes with 1X phosphate-buffered saline (PBS), with the final wash serving as T0.

### 2.4. Measurement of ACE2 Surface Expression in Osteoblasts and MSCs by Flow Cytometry

An amount of 1 × 10⁶ osteoblasts and their precursor cells (MSCs) were washed and incubated with a rabbit primary polyclonal antibody to human ACE2 (PA5.20040, Thermo Fisher Scientific, Waltham, MA, USA) for 1 h on ice. The cells were washed and incubated with a PE-labeled anti-rabbit antibody (ab72465, Abcam, Cambridge, UK). Data were acquired using Full Spectrum Flow Cytometry on the Cytek^®®^ Northern Lights 3000™ (Cytek Biosciences Inc., Fremont, CA, USA) and analyzed with FlowJo v10.6.2 (Ashland, Wilmington, DE, USA).

### 2.5. Detection and Quantification of SARS-CoV-2 Genomic RNA

RNA was extracted from culture supernatants using the Chemagic™ Viral DNA/RNA kit special H96 on the automated Chemagic™ 360 instrument (PerkinElmer, Waltham, MA, USA). After quantification using a NanoDrop™ (Thermo Fisher Scientific), RNA was normalized before performing SARS-CoV-2 RNA detection with RT-qPCR (DisCoVery SARS-CoV-2 RT-PCR Detection Kit Rox), targeting ORF1ab and N viral genes as per the manufacturer’s instructions. To quantify the viral load in culture supernatants, Ct values were interpolated against a standard curve. This curve was generated using Ct values derived from PCR amplification of samples containing serial dilutions of a quantified SARS-CoV-2 positive RNA control (GISAID EPI_ISL_420600).

### 2.6. Detection of Subgenomic (sg) SARS-CoV-2 RNA

For sgRNA, we combined a forward primer in the leader sequence (5′-CGATCTCTTGTAGATCTGTTCTC-3′) with the nucleocapsid 2 (N2) target reverse primer 5′-GGTGAACCAAGACGCAGTAT-3′ and probe N2_P: 5′-FAM-CGATCAAAACAACGTCGGCCCC-BHQ1-3′ as was previously described [14]. PCR reactions were performed using the TaqMan Fast Virus 1-Step Master Mix (Applied Biosystems, Waltham, MA, USA).

### 2.7. Cell Death and Mitochondrial Reactive Oxygen Species (ROS) Production

Cell death was evaluated with Ghost Dye Violet450 (Tonbo^®®^, Cytek Bioscienes Inc., Fremont, CA, USA) with cells exposed to freeze–thaw cycles as the positive control.

Mitochondrial reactive oxygen species (mROS) were measured using 5 μM MitoSOX™ (Thermo Fisher Scientific) staining for 30 min. Cells stimulated with 10 μM rotenone (Sigma-Adrich) were used as a positive control. Data were acquired using Full Spectrum Flow Cytometry Cytek^®®^ Northern Lights 3000™ (Cytek Biosciences Inc.) and analyzed with FlowJo.v10.6.2 (Ashland, Wilmington, DE, USA).

### 2.8. UV-C Irradiation for SARS-CoV-2 Inactivation

To evaluate SARS-CoV-2 inactivation, a UV-C light tube emitting at 253.7 nm with an intensity of 500 μW/cm^2^ was positioned 30 cm above culture plates containing the virus (5 mL, 5 × 10⁴ TCID₅₀/mL in 10 cm dishes). The plates were exposed to UVC irradiation for 60 s. After the exposure, the virus titer was assessed using a TCID₅₀ assay. For this, Vero E6 cells (2 × 10⁴ cells per well) were plated in 96-well plates and infected with 100 μL of serially diluted virus-containing medium prepared in ten-fold steps. Each dilution was tested in 8 replicates. The plates were incubated at 37 °C for 3 days, and the viral titer was determined by observing and quantifying the cytopathic effects (CPE) caused by the virus [15,16].

### 2.9. Evaluation of SARS-CoV-2 Infectious Particles

To evaluate the release of infectious SARS-CoV-2 particles, Vero E6 cells were used. Vero cells were seeded in 96-well plates and exposed to the culture supernatants under investigation for 1 h at 37 °C. After this incubation period, the supernatants were removed, and the cells were provided with fresh DMEM containing 2% FBS. The plates were then incubated for an additional 3 days at 37 °C. The infectious titer of each supernatant was determined by the TCID50 assay and expressed as plaque-forming units per mL (PFU/mL).

### 2.10. Cellular mRNA Preparation and RT-qPCR

Real-time PCR was carried out with a SYBR green as a DNA-binding fluorescent dye using a StepOne Real-Time PCR System (Applied Biosystems). The following primers pair were used: β-actin sense 5- CCTGGCACCCAGCACAAT-3, antisense 5-CGGGATCCACACGGAGTACT-3; Runt-related transcription factor (RUNX)2 sense 5- GGAGTGGACGAGGCAAGAGTTT-3, antisense 5- AGCTTCTGTCTGTGCCTTCTGG-3; IFNβ1 Sense 5- ACGCCGCATTGACCATCTAT-3, antisense 5- GTCTCATTCCAGCCAGTGCT-3; peroxisome proliferator-activated receptor (PPAR)γ sense 5- GGCCGCAGATTTGAAAGAAG-3, antisense 5- GTTTGAGAAAATGGCCTTGTTGT-3; RANKL sense 5- GCCAGTGGGAGATGTTAG-3, antisense 5- TTAGCTGCAAGTTTTCCC-3. Relative transcript levels were calculated using the 2^−ΔΔCt^ method using as normalizer gene β-actin.

### 2.11. Spike (S) Protein Neutralization Assay

Neutralization assays against SARS-CoV-2 were conducted with anti-Spike protein receptor-binding domain (RBD) immunoglobulin F(ab’)2 fragments from hyperimmune equine plasma (Elea Phoenix S.A., Buenos Aires, Argentina) or anti-snakebite F(ab’)2 fragments from hyperimmune equine plasma as the control (Instituto Biológico Argentino S.A.I.C., Buenos Aires, Argentina). For these assays, SARS-CoV-2 (ancestral strain) or the same inoculum of UV-inactivated virus were pre-incubated with neutralizing antibody (or control) at 30 µg/mL for 60 min at 37 °C before use. The concentration used was able to neutralize the infectivity of an inoculum of 1 × 10⁶ PFU/mL, as evaluated in Vero E6 cells

### 2.12. Evaluation of Osteogenic Differentiation

#### 2.12.1. Alkaline Phosphatase (ALP) Activity

ALP staining was performed after 7, 14, and 21 days of osteoblast differentiation using a BCIP (5-Bromo-4-chloro-3-indolylphosphate)-NBT (nitroblue tetrazolium) solution (Sigma-Aldrich) following the manufacturer’s protocol. Briefly, cells were incubated with the BCIP-NBT substrate for 10 min in the dark at room temperature, and the reaction was terminated by washing with distilled water. For ALP activity quantification, cells were lysed in 0.1 M Tris buffer containing 0.5% Triton X-100. Lysates with 2 mg of total protein were incubated with p-nitrophenylphosphate (pNPP) for 10 min at 37 °C. The reaction was stopped by adding 0.5 M NaOH, and absorbance at 420 nm was measured using a microplate reader.

#### 2.12.2. Assessment of Calcium Deposition by Alizarin Red S Staining

After 7, 14, and 21 days of osteoblast differentiation, cells were fixed in 4% PFA to evaluate calcium deposition. Afterward, the culture was stained by 2% (*w*/*v*) Alizarin Red S and examined under a light microscope. To perform quantitative analysis, cell monolayers were detached (10% [*v*/*v*] acetic acid; at 85 °C for 10 min). Supernatants were neutralized by adding 10% (*v*/*v*) ammonium hydroxide. A microplate reader was used to measure absorbance at 405 nm.

#### 2.12.3. Assessment of Collagen Deposition by Sirius Red Staining

Sirius Red (Sigma-Aldrich, Argentina) was used to evaluate the collagen deposition at 7, 14, and 21 days post-differentiation. Before fixation, the cells were stained with Sirius Red dye reagent in 0.1% saturated aqueous picric acid and analyzed by light microscopy. For quantitative evaluation, the formed material was digested in 0.2 mL of 0.1 N sodium hydroxide, and the optical density (OD) was measured at 550 nm in a microplate reader.

### 2.13. Evaluation of Adipocyte Differentiation Through Lipid Droplet Accumulation Analysis

The differentiation of adipocytes was assessed by the accumulation of lipid droplets measured by Bodipy 493/503 (Life Technologies). Cells were grown on 24-well plates, fixed with 10% formalin for 1 h and permeabilized with 0.3% Triton X100. Then, lipid droplets were stained with 1 µg/mL Bodipy 493/503 (Invitrogen, Waltham, MA, USA). Nuclear counterstaining was performed with DAPI (Thermo Scientific). Coverslips were mounted with PBS-glycerin (9:1 *v*/*v*) and analyzed using a Zeiss LSM 800 confocal microscope. Quantification involved analyzing ten microscopic fields per well from three wells per experimental condition.

### 2.14. Statistical Analysis

Multiple comparisons between all pairs of groups were performed using Tukey’s test, while comparisons between two groups were conducted using the Mann–Whitney U test. Each experiment was carried out in triplicate with different culture preparations on five independent occasions. Data are presented as mean ± SD. Statistical significance is indicated as follows: *p* < 0.01 is represented as * *p* < 0.01, ** *p* < 0.005, *** *p* < 0.0005, **** *p* < 0.0001.

## 3. Results

### 3.1. Osteoblasts Are Permissive for Productive SARS-CoV-2 Replication

**Figure 1:**
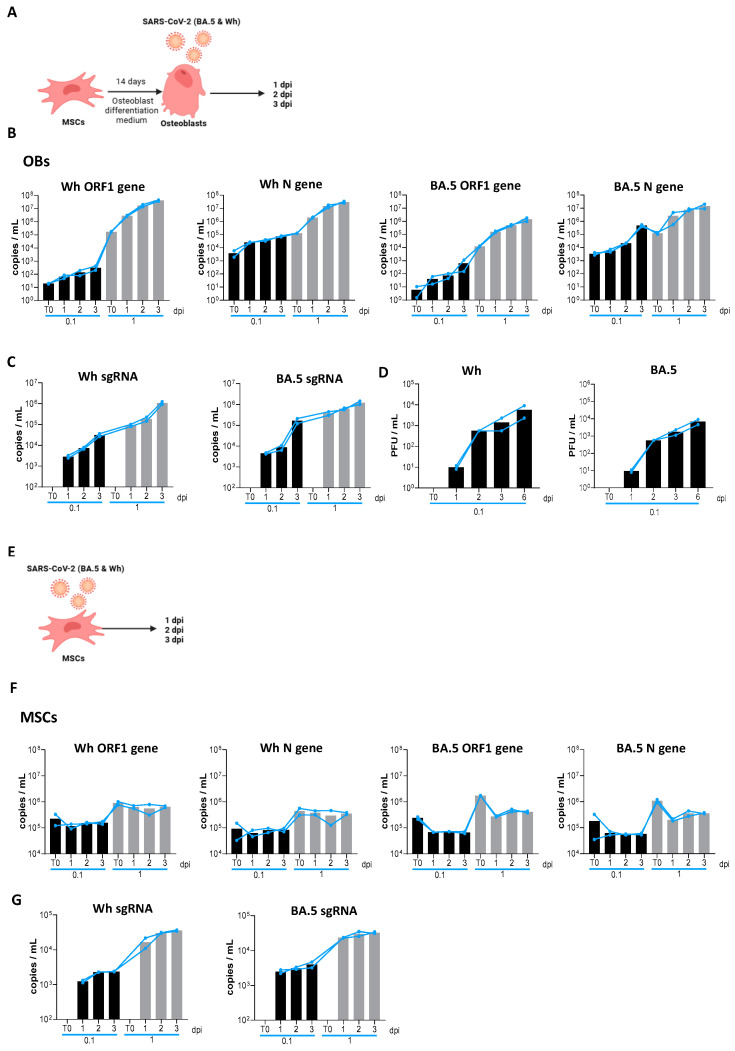
SARS-CoV-2 replication kinetics. (**A**) Experimental timeline schedule for differentiated osteoblasts (OBs). (**B**) Kinetics of SARS-CoV-2 replication in differentiated osteoblasts infected with the ancestral strain (Wh; left) and Omicron strain (BA.5; right) at a multiplicity of infection (MOI) of 0.1 (black bars) and 1 (shadowed bars), measured by mRNA levels of ORF1ab and nucleocapsid (N) in culture supernatants via RT-qPCR. (**C**) Kinetics of transcription of SARS-CoV-2 subgenomics (sg) RNA in differentiated osteoblasts infected with the ancestral strain (Wh; left) and Omicron strain (BA.5; right) at a multiplicity of infection (MOI) of 0.1 and 1, measured by mRNA levels of nucleocapsid 2 (N2). (**D**) SARS-CoV-2 titration (Wh and BA.5 strains) in culture supernatants from differentiated osteoblasts, reported as the number of plaque-forming units per mL (PFU/mL). (**E**) Experimental timeline schedule for mesenchymal stem cells (MSCs). (**F**) Kinetics of SARS-CoV-2 replication in mesenchymal stem cells (MSCs), measured as described in panel B. (**G**) Kinetics of transcription of SARS-CoV-2 subgenomics (sg) RNA in mesenchymal stem cells (MSCs) as described for panel C. dpi: days postinfection. Data are expressed as mean ± SD from four independent experiments.

The experimental timeline, illustrated in Figure 1A, involved exposing osteoblasts differentiated from MSCs to two SARS-CoV-2 variants—ancestral (Wh) and Omicron (BA.5). MSCs were first differentiated into osteoblasts and then infected with the Wh and BA.5 variants at multiplicities of infection (MOI) of 0.1 and 1, corresponding to low and high viral inputs, respectively. Viral load was assessed in culture supernatants at 1, 2, and 3 days post-infection (dpi) using RT-qPCR targeting the N-gene and ORF-1a-gene. Additionally, the viral load was measured in the final wash following inoculum exposure as a baseline control (T0).

Viral loads in culture supernatants were measured at 1, 2, and 3 days post-infection (dpi) using RT-qPCR targeting the N and ORF-1a genes. The results revealed a progressive increase in viral copies from 1 to 3 dpi, with the magnitude of this increase depending on the MOI (Figure 1B), with the presence of sgRNA (Figure 1C). Furthermore, an assay to detect infectious viral particles confirmed de novo production of SARS-CoV-2 particles (Wh and BA.5) in the culture supernatants, persisting up to 3 dpi (Figure 1D). Viral infection did not modify cell viability.

In summary, these findings demonstrate that exposure to the ancestral (Wh) and Omicron (BA.5) SARS-CoV-2 variants leads to productive infection in osteoblasts derived from MSCs. This highlights osteoblasts’ susceptibility and permissiveness to SARS-CoV-2 and underscores the potential implications for bone health during infection.

### 3.2. SARS-CoV-2 Causes an Abortive Infection in MSCs

The experimental timeline, shown in Figure 1E, involved exposing mesenchymal stem cells (MSCs), the precursor cells to osteoblasts, to two SARS-CoV-2 variants—ancestral (Wh) and Omicron (BA.5)—at multiplicities of infection (MOI) of 0.1 and 1. Viral load was assessed in culture supernatants as was described in point 3.1.

For both variants (Wh and BA.5), no significant differences in viral load (genomic RNA) were observed between T0 and 1, 2, or 3 dpi. (Figure 1F). Viral exposure did not modify cell viability. Further analysis of infectious viral particles in supernatants revealed no detectable production of de novo viral particles for either the Wh or BA.5 variant throughout the experiment, but similar levels of synthesizing viral sgRNA were detected by RT-qPCR. Thus, despite being susceptible to infection and initiating viral gene expression by synthesizing viral sgRNA (Figure 1G), MSCs were unable to produce infectious virus particles, as demonstrated by assays designed to detect infectious viral particles, suggesting an abortive infection.

### 3.3. Expression of ACE2 in Precursor Cells and Differentiated Osteoblasts

**Figure 2:**
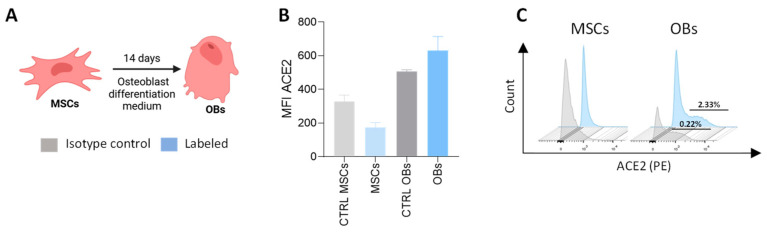
ACE2 expression in mesenchymal stem cells (MSCs) and osteoblasts (OBs). (**A**) Experimental timeline. (**B**) Analysis of ACE2 surface expression in mesenchymal stem cells (MSCs) and differentiated osteoblasts (OBs) using flow cytometry. (**C**) Representative flow cytometry histograms illustrating ACE2 surface expression, as described in panel B. Data are expressed as mean ± SD from three independent experiments.

The susceptibility of osteoblasts and their precursor cells to SARS-CoV-2 infection and replication is directly influenced by the expression of ACE2, the key receptor utilized by the virus for cellular entry [17,18]. Therefore, we assessed the expression of ACE2 on the surface of MSCs and differentiated osteoblasts. Our results revealed that osteoblasts express higher, although not statistically significant, levels of ACE2 compared to cells labeled with the isotype control antibody. In contrast, their precursor cells showed no detectable levels of ACE2 surface expression (Figure 2). Given the low levels of expression in osteoblasts (OB), it is plausible that alternative ACE2-independent entry mechanisms are involved in the infection of these cells [19].

### 3.4. SARS-CoV-2 Inhibits Osteoblast Differentiation and Function

**Figure 3:**
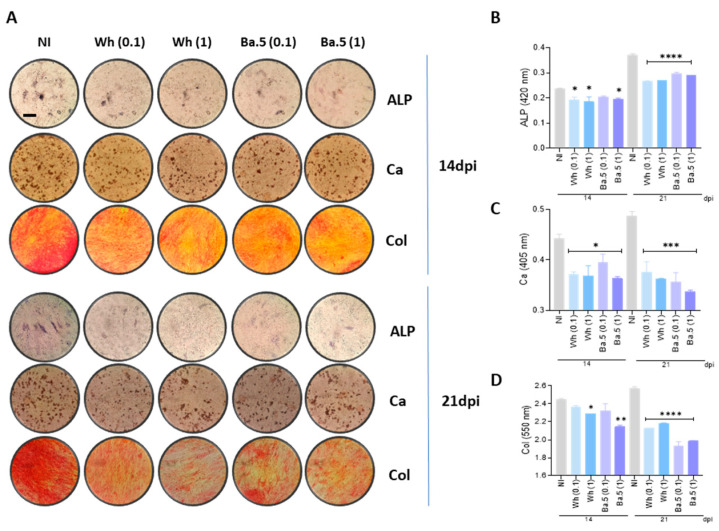
Effect of Wh and BA.5 SARS-CoV-2 variants on osteoblast differentiation. Mesenchymal stem cells (MSCs) were infected with the ancestral strain (Wh) or the Omicron strain (BA.5) at a multiplicity of infection (MOI) of 0.1 or 1, and then cultured in osteoblast differentiation medium. (**A**) Representative microscopy images showing alkaline phosphatase (ALP) activity detected by BCIP-NBT substrate deposition, calcium (Ca) deposition visualized by Alizarin Red S staining, and collagen (Col) deposition identified by Sirius Red staining at 14 days post-infection (dpi) (upper set of images) and 21 dpi (lower set of images). (**B**–**D**) Spectrophotometric quantification of ALP activity (**B**), calcium deposition (**C**), and collagen deposition (**D**). NI: noninfected (fully differentiated positive control); dpi: days post-infection. Ten microscopic fields per condition were quantified for each experiment. Scale bar: 100 µm. Data are expressed as mean ± SD from three independent experiments. * *p* < 0.01, ** *p* < 0.005, *** *p* < 0.0005, **** *p* < 0.0001 vs. NI.

MSCs were infected with two different virus variants of SARS-CoV-2 (Wh and BA.5), and then cultured in osteoblast differentiation medium to observe effects caused by SARS-CoV-2 on osteoblast differentiation and function. Osteoblasts are highly specialized cells that play an active part in the synthesis and upkeep of the bone. ALP is already recognized as a marker and hence was analyzed for activities from day 14 and further from day 21 in general after differentiation by the BCIP-NBT staining. At the same time point, calcium-rich deposits had been assayed by Alizarin Red S staining to study mineralization, and Sirius Red staining measured collagen deposition. Data demonstrated that Wh and BA.5 SARS-CoV-2 variants significantly inhibited ALP activity, collagen, and calcium deposits at 14 and 21 days after differentiation compared to fully differentiated non-infected cells. However, no dose-dependent response was identified in the inhibitory effect of SARS-CoV-2 on osteoblast differentiation, as indicated in Figure 3. Collectively, these results indicate that SARS-CoV-2 exposure impairs osteoblast differentiation and disturbed matrix deposition, which may lead to compromised bone formation.

### 3.5. SARS-CoV-2-Abortively Infected MSCs Depict Redox Imbalance but Preserve Viability

**Figure 4:**
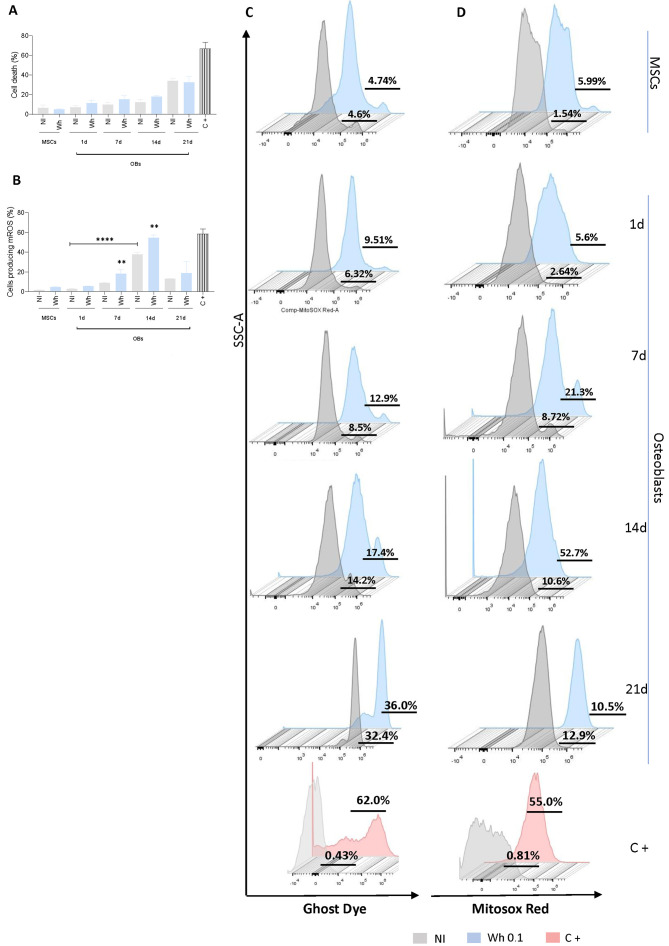
Effects of SARS-CoV-2 on cell viability and mROS production. (**A**) Assessment of cell viability, shown as the percentage of cells stained with Ghost Dye Violet450, following infection with the ancestral SARS-CoV-2 variant (Wh) at a MOI of 1, in MSCs and at four distinct time points: 1, 7, 14, and 21 days post-infection (dpi) during osteoblast differentiation. (**B**) Quantification of mitochondrial reactive oxygen species (mROS) production, expressed as the percentage of cells stained with MitoSOX Red, measured by flow cytometry in MSCs and at the same time points during osteoblast differentiation. (**C**) Representative flow cytometry histograms of Ghost Dye staining at the time points indicated in panel A. (**D**) Representative flow cytometry histograms of MitoSOX staining, illustrating mROS levels at the time points indicated in panel B. NI: noninfected cells. dpi: days post-infection. mROS: mitochondrial reactive oxygen species. SARS-CoV-2: severe acute respiratory syndrome coronavirus 2. MSCs: mesenchymal stem cells. NI: non-infected. C+: positive control. Data are expressed as mean ± SD from three independent experiments. ** *p* < 0.005 vs. NI, **** *p* < 0.0001.

Even in cases of abortive infection, the response of MSCs can be triggered, leading to significant biochemical and morphological changes. To ensure that cell-related parameters are comparable between virus-infected and control cells, cell death was measured using ghost dye. As shown in Figure 4A,C, cell viability remained preserved after exposure to the Wh SARS-CoV-2 strain in MSCs and during osteoblast differentiation up to 21 dpi.

Excessive reactive oxygen species (ROS) have a detrimental impact on osteoblast differentiation, proliferation, and survival [20,21]. ROS production increased during osteoblast differentiation at 7 and 14 days post-differentiation, with this increase being more pronounced in cells exposed to SARS-CoV-2 (Figure 4B,D). Therefore, it may be suggested that ROS can play a pivotal role in inhibiting the differentiation of osteoblasts after exposure to the SARS-CoV-2 virus.

### 3.6. SARS-CoV-2 Modulates RUNX2 and PPARγ Transcription

**Figure 5:**
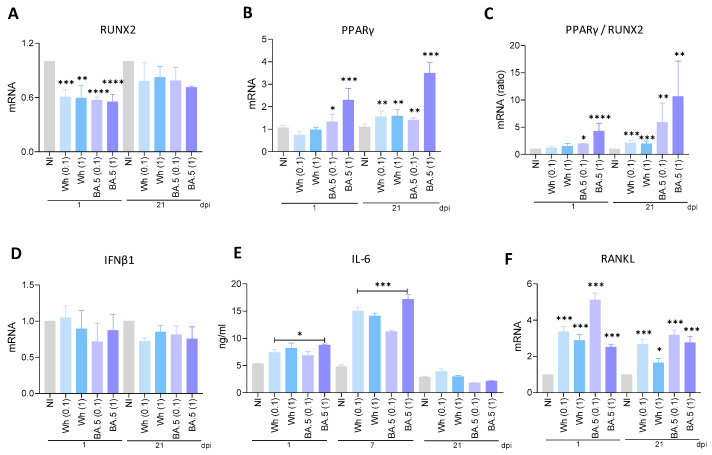
SARS-CoV-2 modulates RUNX2, PPARγ, IFNβ1, IL-6, and RANKL expression during osteoblast differentiation. Mesenchymal stem cells (MSCs) were infected with the ancestral strain (Wh) or the Omicron strain (BA.5) at a multiplicity of infection (MOI) of 0.1 or 1, and then cultured in osteoblast differentiation medium. The transcription levels of RUNX2, PPARγ, IFNβ1, and RANKL were assessed by RT-qPCR at 1 and 21 dpi. IL-6 expression was measured in culture supernatants by ELISA at 1, 7, and 21 dpi. Panels show the following: RUNX2 (**A**), PPARγ (**B**), PPARγ/RUNX2 ratio (**C**), IFNβ1 (**D**), IL-6 (**E**), and RANKL (**F**). NI: noninfected; dpi: days post-infection. Data are expressed as mean ± SD from three independent experiments. * *p* < 0.01, ** *p* < 0.005, *** *p* < 0.0005, **** *p* < 0.0001 vs. NI.

RUNX2 and PPARγ are transcription factors well known as key regulators of MSC differentiation toward osteoblasts and adipocytes, respectively. Their activity is mutually exclusive and tightly controlled since they determine lineage commitment in MSCs [22]. To investigate if SARS-CoV-2 modulates RUNX2 and PPARγ transcription, MSCs were infected with two SARS-CoV-2 strains (Wh and BA.5). By RT-qPCR, we measured RUNX2 and PPARγ transcription levels at 1 and 21 days after osteoblast differentiation. Both SARS-CoV-2 strains strongly inhibited RUNX2 transcription at 1 dpi. At 21 dpi, however, SARS-CoV-2-infected cells expressing Wh or BA.5 failed to show significant suppression of RUNX2 transcription relative to mock cells (Figure 5A). The evaluation of the transcriptional expression of PPARγ evidenced that at both 1 and 21 days of differentiation after infection, PPARγ was upregulated by SARS-CoV-2 BA.5. It is noteworthy to point out the Wh strain upregulated PPARγ transcription at 21 dpi only (Figure 5B). The ratio between PPARγ/RUNX2 has consistently increased with both SARS-CoV-2 strains only at 21 dpi; only the BA.5 strain, however, increased it at 1 dpi, as shown in Figure 5C. Our findings suggest that SARS-CoV-2 may tilt MSCs differentiation towards adipogenesis at the expense of osteogenesis and thus interfere with bone and metabolic homeostasis.

### 3.7. SARS-CoV-2 Variants Were Unable to Promote IFNβ1 Transcription

IFNβ1 has been recognized for its key role in the antiviral immune response against viral infections, including SARS-CoV-2 [23]. However, it may be involved in regulating osteoblast differentiation from MSCs, too [24]. Along this line, IFNβ1 has been found to interfere with the osteogenic differentiation process of MSCs at least in part by modulating some key transcription factors critical to the process, including RUNX2, crucial for osteoblast commitment [25]. To investigate whether SARS-CoV-2 was capable of modulating IFNβ1 expression in MSCs during osteoblast differentiation, MSCs were infected with SARS-CoV-2 and then further cultured in osteoblast differentiation medium. IFNβ1 levels were quantified at 1 and 21 dpi. As shown in Figure 5D, infection of MSCs with the Wh and BA.5 virus strains did not modulate IFNβ1 transcription levels at either 1 or 21 dpi compared to non-infected cells. These results strongly support that IFNβ1 does not seem to be involved in the inhibition of osteoblast differentiation following SARS-CoV-2 infection.

### 3.8. Wh and BA.5 SARS-CoV-2 Strains Induce IL-6 Secretion During Osteoblast Differentiation

IL-6 exerts diverse effects on bone turnover, including its potential to contribute to bone loss by inhibiting Runx2 and consequently suppressing matrix mineralization [26,27]. IL-6 secretion was significantly increased in SARS-CoV-2-infected osteoblasts compared to control cells at 1 dpi but returned to basal levels by 21 dpi (Figure 5E). Therefore, IL-6 levels were measured at 7 dpi and observed that the levels remained higher in SARS-CoV-2-infected cells than in uninfected controls (Figure 5E). Additionally, these levels were elevated compared to those measured at 1 dpi. These results suggest that IL-6 may act as a soluble factor involved inhibiting osteoblast differentiation. IL-6 exerts several paradoxical effects on bone resorption, which may either directly contribute to bone loss through its inhibitory action on Runx2 and matrix mineralization [26,27] or exert protective functions [28,29]. IL-6 expression was strongly upregulated in SARS-CoV-2-positive osteoblasts compared with uninfected control cells at 1 dpi, returning to the level measured in control cells by 21 dpi (Figure 5E). Thus, we decided to quantify the level of IL-6 at 7 dpi and detected higher IL-6 in SARS-CoV-2-infected cells than in uninfected controls (Figure 5E). This level is also higher when compared with that detected at 1 dpi. This may suggest that IL-6 could be one of the soluble factors involved in inhibiting the differentiation of osteoblasts.

### 3.9. SARS-CoV-2 Induces RANKL Expression in Osteoblasts

Activated by IL-6, osteoblasts and their precursor cells produce pro-osteoclast mediators like RANKL [27,28]. Based on the latter, experiments were conducted to test whether SARS-CoV-2 Wh and BA.5 induced RANKL expression. Therefore, our data showed that SARS-CoV-2 Wh and BA.5 were able to upregulate mRNA transcription of RANKL at 1 and 21 dpi to controls (Figure 5F). These results support the hypothesis that IL-6 acts as a soluble factor inducing RANKL expression, enhancing subsequently osteoclastogenesis, and thus indirectly contributing to the inhibition of osteoblast differentiation.

### 3.10. UV-Inactivated SARS-CoV-2 Was Able to Inhibit Osteoblast Differentiation

**Figure 6:**
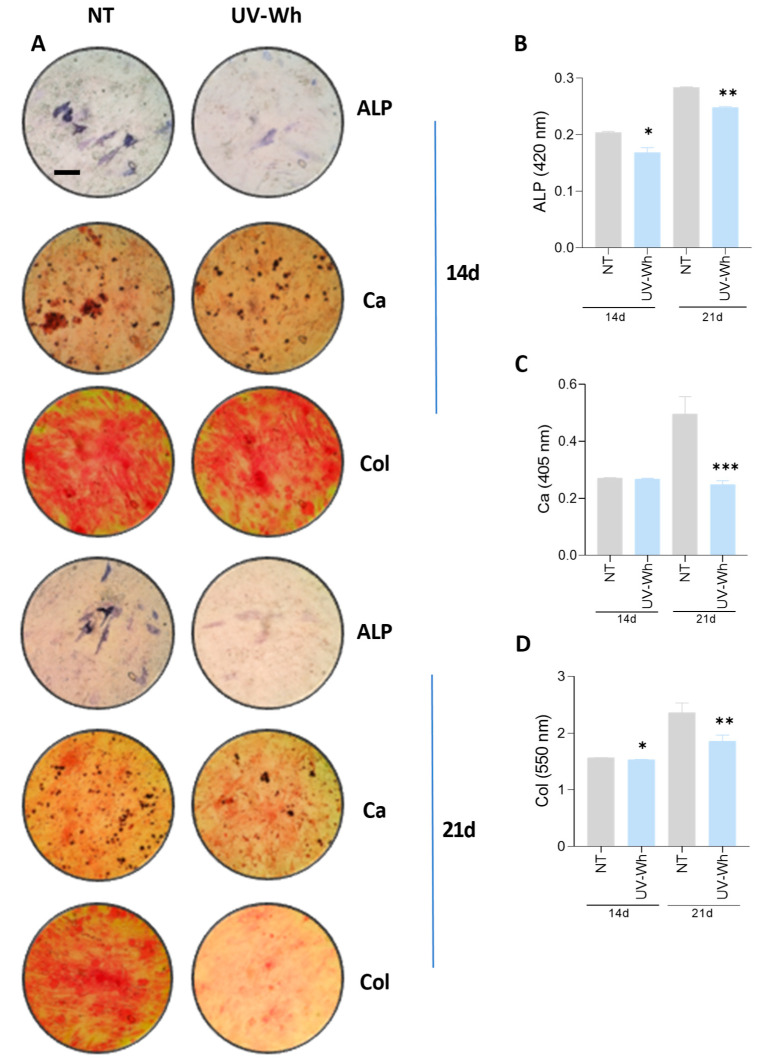
UV-inactivated SARS-CoV-2 inhibits osteoblast differentiation. Effect of UV-inactivated SARS-CoV-2 Wh strain on osteoblast differentiation. (**A**) Representative microscopy images showing alkaline phosphatase (ALP) activity (visualized by BCIP-NTB substrate deposition), calcium deposition (Ca, stained with Alizarin Red S), and collagen deposition (Col, stained with Sirius Red) at 14 and 21 days post-infection (dpi). (**B**–**D**) Spectrophotometric quantification of ALP activity (**B**), calcium deposition (**C**), and collagen deposition (**D**). NI: noninfected; d: days post-stimulation. Ten microscopic fields per condition were quantified for each experiment. Scale bar: 100 µm. Data are expressed as mean ± SD from three independent experiments. * *p* < 0.01, ** *p* < 0.005, *** *p* < 0.0005 vs. NI.

To determine whether the response to SARS-CoV-2 was linked to viral infectivity, MSCs were exposed to UV-inactivated SARS-CoV-2 (Wh) at an equivalent amount to MOI of 0.1 during the osteoblast differentiation process. By 14 and 21 days post-differentiation, ALP activity and also calcium and collagen deposition were measured. Our findings indicated that UV-inactivated virus significantly inhibited osteoblast differentiation, as shown by the decrease in ALP activity, calcium deposition, and collagen deposition in Figure 6. Inactivated SARS-CoV-2 virus can be obtained by UV inactivation, retaining antigenic properties of the native virus, as well as morphology of the viral particles [30,31]. Thus, this finding indicated that a structural viral factor has the potential to drive osteoblast differentiation.

### 3.11. Spike–S-Protein Has a Role in the Inhibition of Osteoblast Differentiation

**Figure 7:**
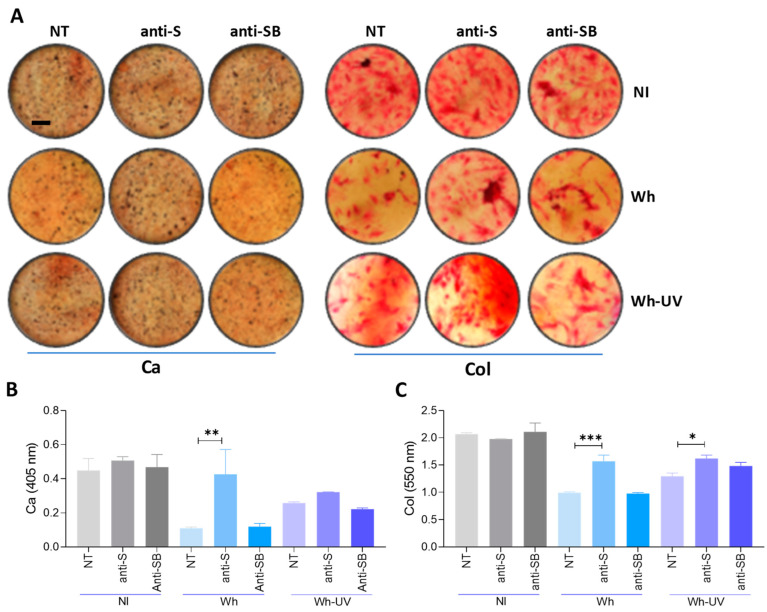
The Spike (S) glycoprotein inhibits osteoblast differentiation. Mesenchymal stem cells (MSCs) were infected with the ancestral variant (Wh) or UV-C-inactivated virus at a multiplicity of infection (MOI) of 0.1. Infectious virus or UV-C-inactivated virus was pre-incubated for 1 h at 37 °C with an anti-spike glycoprotein antibody (anti-S) or an anti-snakebite antibody as a control (anti-SB). The cells were then cultured in osteoblast differentiation medium. (**A**) Representative microscopy images showing calcium deposition (stained with Alizarin Red S) and collagen deposition (stained with Sirius Red) at 21 days post-infection (dpi). (**B**,**C**) Spectrophotometric quantification of calcium deposition (**B**) and collagen deposition (**C**). NI: non-infected; NT: non-treated. Ten microscopic fields per condition were quantified for each experiment. Scale bar: 100 µm. Data are expressed as mean ± SD from three independent experiments. * *p* < 0.01, ** *p* < 0.005, *** *p* < 0.0005, vs. NI.

Experiments are conducted to deduce the influence of the S glycoprotein on the differential stages of osteoblast precursors. In carrying out this task, the neutralization experiments are undertaken with an anti-SARS-CoV-2 spike protein receptor-binding domain-neutralizing antibody, while the control is an anti-snakebite antibody. For these experiments, SARS-CoV-2 [ancestral strain, multiplicity of infection (MOI) 0.1] or an equivalent inoculum of UV-inactivated virus was pre-incubated for 1 h at 37 °C with neutralizing antibody (or control) at a concentration of 30 µg/mL before use. Neutralization of the S glycoprotein reduced the ability of infective SARS-CoV-2 and UV-inactivated virus to suppress calcium and collagen deposition, whereas the control antibody did not (Figure 7). These results suggest that the S glycoprotein plays an important role in SARS-CoV-2-induced inhibition of osteoblastogenesis.

### 3.12. SARS-CoV-2 Could Not Modulate Adipocyte Differentiation

**Figure 8:**
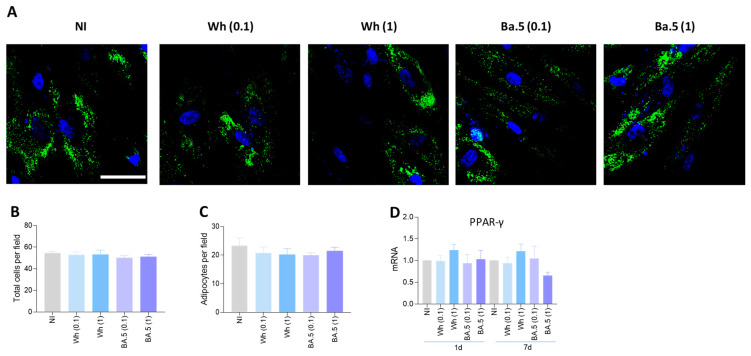
Effect of Wh and BA.5 SARS-CoV-2 strains on adipocyte differentiation. Mesenchymal stem cells (MSCs) were infected with the ancestral strain (Wh) or the Omicron strain (BA.5) at a multiplicity of infection (MOI) of 0.1 or 1, and then cultured in adipocyte differentiation medium. (**A**) Representative images of lipid droplets stained with Bodipy 493/503 at 7 days post-differentiation. (**B**,**C**) Quantification of the experiment shown in panel A: number of cells per field (**B**) and percentage of adipocytes per field (**C**). (**D**) PPARγ expression was measured by RT-qPCR at 1 and 7 days post-infection (dpi). NI: non-infected; dpi: days post-infection. Ten microscopic fields per condition were quantified for each experiment. Scale bar: 50 µm. Data are expressed as mean ± SD from three independent experiments.

A pathologic reduction in bone mineral density has been demonstrated to be associated with fat accumulation [32]. Given that both osteoblasts and adipocytes originate from MSCs, the predisposition of MSCs to differentiate into adipocytes rather than into osteoblasts is thought to be one contributing factor to bone loss. To investigate whether SARS-CoV-2 influences the process of adipocyte differentiation, MSCs were infected with the Wh and BA.5 SARS-CoV-2 strains in the presence of adipocyte differentiation media. Differentiated adipocytes were detected by staining the lipid droplets with Bodipy 493/503. The infection of MSCs with SARS-CoV-2 did not alter the process of adipocyte differentiation, since the amount of differentiated adipocytes did not change in comparison to the uninfected cells (Figure 8A–C). The commitment of MSCs toward the osteoblast or adipocyte lineage is dictated by specific transcriptional regulators [32,33]. Among these, peroxisome proliferator-activated receptor gamma (PPARγ) is a critical regulator of adipogenesis [34], and its expression represses osteoblast differentiation by inhibiting Runx2 transcription [35]. In line with the absence of enhanced adipocyte differentiation, the Wh and BA.5 SARS-CoV-2 strains failed to trigger an upregulation of PPARγ expression (Figure 8D). These results suggest that SARS-CoV-2 exposure does not influence the commitment of MSCs toward the adipocyte lineage.

## 4. Discussion

COVID-19 has been presented with a wide range of clinical manifestations, from asymptomatic infection to severe conditions such as ARDS and multiple organ dysfunction [36,37]. Though neurological, pulmonary, renal, vascular, and cardiac complications have been widely studied, the musculoskeletal system, particularly bones and joints, has only recently come into the spotlight. It is important to recognize the potential effects that respiratory diseases, including COVID-19, have on bone health. These can contribute to greater bone loss and fragility, thereby increasing vulnerability to life-threatening comorbidities.

Bone loss progresses with age and increases the risk for diseases like osteoporosis, and compromises health and quality of life [38]. Herein, the authors show, using ancestral and Omicron SARS-CoV-2 variants, abortive infection of SARS-CoV-2 in osteoblast precursor cells (MSCs), whereas both strains productively infected and replicated in differentiated osteoblasts, producing de novo viral RNA and releasing virions. This was despite low, statistically insignificant increases in ACE2 expression in differentiated osteoblasts compared to their precursor cells. Although ACE2 is the main receptor for SARS-CoV-2, there are several reports on potential alternative receptors: tyrosine-protein kinase receptor UFO (AXL), CD4, CD147, KREMEN1, HSPGs, sialic acids, TMEM106B, NPC1, and the transferrin receptor [39,40,41,42,43,44,45]. Their role in the infection of osteoblasts and precursor cells remains to be determined.

In the present study, exposure of osteoblast precursors to the Wh and BA.5 strains of SARS-CoV-2 inhibited osteoblast differentiation, as evidenced by suppression of ALP activity and downregulation of calcium and collagen deposition. This decrease in osteoblast differentiation was not because of a loss of viability in the differentiated osteoblasts or their precursor cells, as cell viability remained comparable between virus-exposed and non-exposed cells.

ROS are highly reactive molecules that may activate the signaling pathways that promote osteoblast differentiation by activating key transcription factors such as RUNX2 and Osterix driving this process [46,47]. On the other hand, high levels of ROS, or oxidative stress, inhibit osteoblast differentiation by oxidizing RUNX2, leading to its degradation [48,49,50]. In line with this, according to conditions of Wh and BA.5 SARS-CoV-2 infection, the remarkably increased MSCs at both 7 and 14 dpi provided evidence for the view that the elevation of ROS can potentially contribute to the SARS-CoV-2-suppressing effect on the osteoblast differentiation in MSCs.

Reciprocal regulation of RUNX2 with PPARγ is extensively sophisticated, including the actions of Wnt-positive regulating RUNX2 expression together with the repression of PPARγ. This creates an explicit feedback loop that equips these transcription factors to balance their expression [51,52,53]. Concordant with the above concept and in support of such observations from cellular lineage, a significant increase in the PPARγ/RUNX2 ratio after infection with SARS-CoV-2 was identified in precursor cells and differentiated osteoblasts.

Osteoblastogenesis is a differentiation process well orchestrated through complex cytokine networks acting under physiologic and pathophysiologic states [54]. Type I interferons, consisting of a large multi-gene family exhibiting distinct subtypes, each relate to different functional roles regarding their action on osteoblasts. While IFNα enhances the differentiation of osteoblasts, thus contributing to the integrity of the bone matrix, IFNβ inhibits bone remodeling mediated through osteoblasts, emphasizing the opposite effects of interferons on bone homeostasis [55]. An excessive or dysregulated production of type I interferons, as noted in severe SARS-CoV-2 infections, might impair bone remodeling and lead to osteoblast dysfunction and suppression of bone formation, thereby leading to inflammatory bone loss. Furthermore, type I interferons may indirectly influence the activity of osteoclasts and enhance bone resorption, further compromising bone health after infection.

Although elevated expression of IFNβ in this model may be merely the result of production from the infiltrating leukocytes, it has been shown that osteoblasts produce IFNβ [56,57]. Similarly, MSCs have been shown to secrete type I interferons following viral challenge [58]. However, our results indicate that MSCs were not able to induce IFNβ transcription after SARS-CoV-2 infection.

IL-6 is another cytokine that negatively regulates osteoblast differentiation by inhibiting the expression of crucial genes for the process, such as Runx2 and ALP [26]. Following the inhibition of osteoblast differentiation induced by SARS-CoV-2 infection, both variants triggered the production of IL-6. IL-6 has been associated with the over-induction of RANKL and downregulation of osteoprotegerin (OPG), the soluble decoy receptor for RANKL, with a consequent association of both with bone osteolysis [59]. An upsurge in transcription of RANKL associated with IL-6 was produced after SARS-CoV-2 infection.

Although the replication cycle of SARS-CoV-2 is between 7 and 24 h [60], virus-infected cells release viral proteins into the extracellular medium relatively early in the course of infection, typically within 6 to 12 h post-infection [61]. These viral proteins might also change the osteoblastic differentiation potential of MSCs. Accordingly, we show herein that UV-inactivated SARS-CoV-2, whose protein structures remain intact, impairs osteoblast differentiation as efficiently as infectious viral particles. The above finding underlines that the inhibitory effect on osteoblast differentiation is due to a structural component. Furthermore, it was observed that the neutralizing antibody against envelope S glycoprotein could reverse the effect of infective particles or UV-C-inactivated SARS-CoV-2 on osteoblast differentiation, suggesting its role in inhibiting the differentiation process).

Since the differentiation of adipocytes and osteoblasts from MSCs represents two counter-regulated processes, we have assumed that the inhibition of osteoblast differentiation might favor the process of adipocyte differentiation. However, neither the Wh nor BA.5 strain of SARS-CoV-2 was able to influence the process of adipocyte differentiation.

The present study has several limitations: it is performed with MSCs obtained from umbilical cords, not from other sources. Second, the absence of any microenvironment context, such as cell-to-cell interactions or the extracellular matrix, impairs our ability to study the modulation of the observed phenomena. Third, we did not investigate the possible direct and indirect role of immune cells that may infiltrate the tissue. Fourth, some mediators could only be measured at the mRNA level due to technical constraints, limiting our ability to assess their protein expression.

## Data Availability

The raw data supporting the conclusions of this article will be made available by the authors, without undue reservation.

## Abbreviations

The following abbreviations are used in this manuscript:

MSCs	mesenchymal stem cells
α-MEM	α-Minimal Essential Medium
DMEM	Dulbecco’s Modified Eagle Medium
FBS	fetal bovine serum
ATCC	American Type Culture Collection
PBS	phosphate-buffered saline
IBMX	3-isobutyl-1-methylxanthine
mROS	mitochondrial reactive oxygen species
CPE	cytopathic effects
RUNX2	runt-related transcription factor 2
IL-6	interleukin-6
RANKL	receptor activator of nuclear factor kappa B ligand
RBD	receptor-binding domain
COVID-19	Coronavirus Disease 2019
SARS-CoV-2	severe acute respiratory syndrome coronavirus
ALP	alkaline phosphatase
BMD	bone mineral density
α-MEM	α-Minimal Essential Medium
sg	subgenomic
mROS	mitochondrial reactive oxygen species
CPE	cytopathic effects
PFU	plaque-forming units
S	spike
RBD	spike protein receptor-binding domain
BCIP	5-Bromo-4-chloro-3-indolylphosphate
NBT	nitroblue tetrazolium
pNPP	p-nitrophenylphosphate
OD	optical density
PPAR	peroxisome proliferator-activated receptor
